# Exploring the risk of depression and suicide/self-injury in patients receiving GLP-1RAs combined with metformin versus GLP-1RA monotherapy: a real-word analysis of the FAERS database

**DOI:** 10.1530/EC-25-0403

**Published:** 2026-02-25

**Authors:** Wenjun Ji, Ying Ma, Jie Fang, Linwei Chen

**Affiliations:** ^1^ Department of Pharmacy, The Affiliated Taizhou People’s Hospital of Nanjing Medical University, Taizhou, China; ^2^ Department of Pharmacy, Ruijin Hospital, Shanghai Jiao Tong University School of Medicine, Shanghai, China

**Keywords:** depression, suicide/self-injury, GLP-1RAs combined with metformin, FAERS

## Abstract

**Objective:**

This study aimed to assess the latent threat of depression and suicide/self-injury associated with the combination of glucagon-like peptide-1 agonists (GLP-1RAs) and metformin versus GLP-1RAs monotherapy, through analyzing the data from the Food and Drug Administration (FDA) Adverse Event Reporting System (FAERS).

**Methods:**

We systematically searched the FAERS database for reports on the concomitant use of GLP-1RAs and metformin compared with the GLP-1RAs monotherapy in diabetic or obese patients. Reports were categorized on the basis of the Medical Dictionary for Regulatory Activities (MedDRA) terminology. The signal was considered to be significant when the reporting odds ratio (ROR) and lower limit of 95% CI > 1, and information component (IC)025 > 0, in no less than three patients.

**Results:**

The addition of metformin increased the risk of death (38.5 vs 12.4%, *P* < 0.001) and hospitalization – initial or prolonged (13.5 vs 7.2%, *P* = 0.035) attributed to suicide/self-injury in patients who received GLP-1RAs. Furthermore, the combination of GLP-1RAs with metformin was disproportionately linked to a higher incidence of suicide/self-injury (ROR = 50.89, 95% CI: 40.79–63.48, IC025 = 5) and depression (except suicide and self-injury) (ROR = 17.28, 95% CI: 13.65–21.87, IC025 = 3.64) when compared with the GLP-1RAs monotherapy. The addition of metformin was confirmed to have shorter intervals to time-to-onset (TTO) than the GLP-1RAs monotherapy.

**Conclusions:**

The combination of GLP-1RAs and metformin is linked to a higher risk of depression and suicide/self-injury compared with the GLP-1RAs monotherapy.

## Introduction

As one of the largest global health emergencies in the current society, diabetes is characterized by chronic hyperglycemia and multiple complications (1, 2). The number of diabetes patients will reach a scary 537 million by 2030, and ∼90% of these cases will be type 2 diabetes (T2DM) (3). Cardiovascular (CV) complications are the first reason for mortality and morbidity of T2DM (4), with a threefold increase in the death rate of diabetes with coronary artery disease (5). The life expectancy of diabetes patients accompanied by atherosclerotic cardiovascular disease (ASCVD) will decrease by approximately 12–15 years, and the CV risk should be treated as a priority rather than focusing on glucose according to the latest guidelines (6, 7). The therapy of T2DM has transferred the priority from a glucose-centered to a complication-centered approach (8).

In general, societal pressure to conform to idealized body standards is widespread, bringing additional hurdles for individuals with obesity or diabetes. It is believed that negative body image and diminished self-esteem will contribute to an increased risk of depression/suicide. T2DM and depression are interactional. Subba *et al.* found that T2DM had a higher risk of depression than non-T2DM, because T2DM has multiple complications and subsequently decreased life quality (9). At the same time, a study confirmed that patients with depression exhibit a twofold increased risk of suffering from T2DM, probably because depressive state contributes to chronic unhealthy lifestyles, including tobacco use, physical inactivity, and excessive alcohol consumption (10). Furthermore, patients with a longer duration of T2DM exhibited a higher risk of suicidal ideation compared to those with a shorter disease duration (11). Certainly, the severity of T2DM and depression was correlated. A study reported a positive correlation between depression and the level of HbA1c, which means the state of T2DM, because a higher HbA1c tends to accompany a greater risk of diabetes complications (12). Thus, it is important to find an effective and reasonable therapy for T2DM as soon as possible.

Currently, it is crucial to figure out the relationship between antidiabetic medications and depression or suicidality. A meta-analysis involving 2,071 adults revealed significant amelioration of depression symptoms in patients treated with GLP-1RAs in contrast to controls (13). A Japanese study implied a lower depression risk of in T2DM patients treated with DPP-4i (14), which was in accordance with an animal model (15). A case–control study demonstrated that SGLT-2i was associated with significant reductions in depression (16).

Glucagon-like peptide-1 receptor agonists (GLP-1RAs) played a role in the regulation of glycometabolism via stimulating the secretion of insulin by simulating the physiological action of endogenous GLP-1 (17). By decreasing appetite, enhancing satiety, delaying gastric emptying, and hastening the growth of the pancreatic *β* cells, GLP-1RAs benefit diabetic and obese patients. In recent years, GLP-1RAs have received a lot of attention, because of being recommended by the American College of Cardiology (ACC) (18) and the American Diabetes Association (ADA) (19) as a first-line treatment for treating diabetes accompanied by ASCVD or kidney disease.

Despite their efficacy in treating T2DM, GLP-1RAs have raised concerns regarding the potential risk of mental health, with many studies reporting the occurrence of suicidal thoughts among patients (20). O’Neil PM *et al.* reported that liraglutide (0.27%) may induce higher suicidal thoughts than the placebo (0.10%) (21). In addition, a disproportionality analysis published in the JAMA Network Open demonstrated causality between semaglutide and suicidal ideation, which requires clarification urgently (22). Currently, inconsistent evidences have come out on the depression risk of GLP-1RAs. GLP-1RAs were considered to protect against neuroinflammation, a disease which was closely associated with depression (23). Grant *et al.* investigated the mental condition of T2DM patients treated with exenatide, and an improved depression state was found in the exenatide group than in the insulin group (24). A study reported the relationship between liraglutide and depression through involving 36 obese women, and no significant association was found (25). Another study has warned of the psychiatric risk, particularly depression and anxiety related to the use of semaglutide, liraglutide, and tirzepatide (26).

Even so, the relationship between semaglutide and liraglutide use and suicidal ideation/depression was denied by the manufacturer. It should be noted that, in the clinical trials on semaglutide and liraglutide, participants with any prior history of suicidal thoughts and mental disease were excluded (27). The EMA conducted a series of reviews on suicidal ideation, self-injury, and depression related to semaglutide and liraglutide intake due to a range of reports of suicidal thoughts during the use of these medications (28). Meanwhile, a warning regarding suicidal behavior and thoughts in patients taking semaglutide and liraglutide was given by an Icelandic study, providing 150 reports related to GLP-1RA-induced suicide/self-injury to the EMA (29).

Metformin is a core component in complication-centered therapy of T2DM, attributed to its efficacy, safety, and cardiovascular benefits. Until 2020, the European Association for the Study of Diabetes (EASD) and the ADA recommended metformin as the first-line therapy in lowering the glucose of T2DM (30). Another guideline mentioned that metformin should be maintained as part of T2DM therapy if no contraindications are present (31). In individuals with diabetes and preexisting nephropathy, GLP-1RAs are recommended as a first-line treatment for T2DM, with metformin being the first choice for the combination therapy. For patients with coronary artery disease, initial treatment should involve GLP-1RAs to reduce the risk of CVD, and certainly, metformin is the first option when a combination therapy is needed (32).

As AlHussain *et al.* found, metformin could decrease the risk of severe depression by 70% (33), with improving neuroinflammation by elevating agmatine (34). Research also reported a potential protective effect of metformin against depression and anxiety in patients with T2DM (35). However, different opinions were suggested. It was reported that metformin was confirmed to stimulate the secretion of GLP-1, thereby exerting a synergistic effect with GLP-1RAs, which may be responsible for the occurrence of suicide (36).

GLP-1RAs were reported to be related to the risk of depression/suicide. The mechanism may be attributed to the modulation of GLP-1RAs on the central nervous system. Through activating the GLP-1 receptors in brain, GLP-1RAs exert a potent anorexigenic effect. In addition, GLP-1RAs could restrain the reward feedback, diminish the neural basis of pleasure, and cause a profound reward deficit, which is the core symptom of depression.

In 2019, GLP-1RAs remained the recommended first-line therapy, by the European Society of Cardiology (ESC), for diabetic patients with ASCVD or at high CV risk, irrespective of metformin use (7). However, a remarkable point the needs to be noticed is that a clear majority of phase III clinical trials and CVOTs of GLP-1RAs were performed in diabetes administrated with metformin. Therefore, it is impossible to deduce whether analogical CV–renal protection is obtained by GLP-1RAs alone in the absence of metformin.

Although some reports denied the causal relationship between GLP-1RAs and suicidal thoughts/depression in patients with T2DM or obesity (37, 38), treatment with GLP-1RAs is still insecure, because it is usually used in combination with metformin in T2DM. The combination of metformin and GLP-1RAs is extensively applied in T2DM who have suboptimal glycemic control, CV risk, or obesity. The guideline recommended metformin combined with a second antidiabetic drug (such as GLP-1RAs) as the therapy when the HbA1c level exceeded the target by ≥1.5% (39). It is not reasonable to discuss the suicidal risk of GLP-1RAs alone. The suicidal/depression risk of GLP-1RAs warrants further investigation in the presence of metformin.

Compared with the GLP-1RAs monotherapy, the assessment of depression/suicide risk of the combination therapy of GLP-1RAs and metformin warrants greater attention. The underlying mechanism may involve the regulation of the hypothalamic–pituitary–adrenal (HPA) axis, testosterone, and vitamin B12 by metformin. Metformin was associated with depression, and the mechanism may be attributed to the regulation of the HPA axis and testosterone. It is reported that a suicide attempt risk was discovered in males with lower levels of testosterone (40). An additional study suggested that a low plasma total testosterone level produces the behavior of suicide attempts by disrupting the hypothalamic–pituitary–gonadal (HPG) axis (41). It is hypothesized that the weight loss achieved with metformin, by alleviating insulin resistance in the hypothalamus, may elicit significant emotional and psychological reactions, thereby potentially impacting suicidal/self-injury behaviors (42). Furthermore, the deficiency of B12 would result from the long-term use of metformin, with a 15% increased annual risk of vitamin B12 deficiency, and then impair the cognitive function, which is part of the symptomatology of depression (43).

Recently, the proof evaluating the risk of suicide/self-injury is poor. Few studies have indicated a plausible correlation between GLP-1RAs and suicidal thoughts and depression (44, 45), and no article has analyzed the risk in the combination therapy of GLP-1RAs and metformin. This study evaluated the risk of suicide/self-injury and depression in diabetes patients receiving GLP-1RAs combined with metformin versus those receiving GLP-1RAs alone, exploiting the Food and Drug Administration (FDA) Adverse Event Reporting System (FAERS) database.

## Materials and methods

### Data source

Our data were retrieved from the FAERS database, which is an open platform to collect the postmarketing safety data of drugs from the drug manufacturers compulsively and consumers of their own accord. To make certain the suicidal risk of GLP-1RAs combined with metformin, we analyzed the FAERS database from 2014Q1 to 2024Q3 related to eight GLP-1RAs (semaglutide, loxenatide, dulaglutide, albiglutide, tirzepatide, liraglutide, lixisenatide, and exenatide). In addition, the chemical compound of drugs such as ‘LY3298176’ was also used to avoid missing any reports.

### Identification of depression and suicide/self-injury

By retrieving MedDRA 25.0, 12 narrow preferred terms (PTs) involving ‘adjustment disorder with depressed mood’, ‘anhedonia’, ‘decreased interest’, ‘depressed mood’, ‘depression’, ‘depressive symptom’, ‘discouragement’, ‘feeling of despair’, ‘feelings of worthlessness’, ‘major depression’, ‘mixed anxiety and depressive disorder’, and ‘perinatal depression’ were associated with depression (excluding suicide and self-injury), a standard MedDRA query (SMQ). Nine narrow PTs involving ‘completed suicide’, ‘depression suicidal’, ‘intentional overdose’, ‘intentional self-injury’, ‘self-injurious ideation’, ‘suicidal behavior’, ‘suicidal ideation’, ‘suicide attempt’, and ‘suicide threat’ were found to be associated with the SMQ for suicide/self-injury. Moreover, 27 system organ classes (SOCs) were also obtained from the MedDRA, and one PT was connected with more than one SOC.

**Figure 1:**
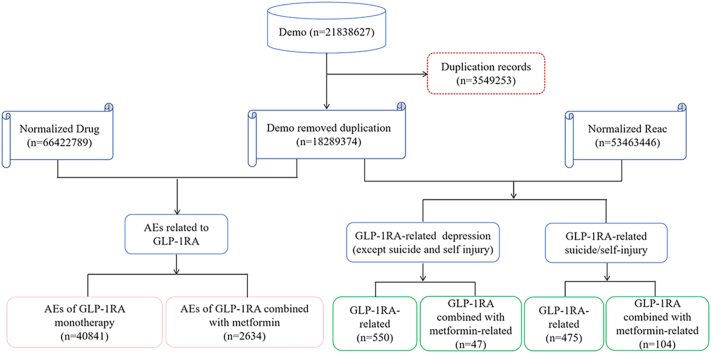
Flow chart of data retrieval process from 2014Q1 to 2024Q3.

The results were standardized through data cleaning and normalized techniques. The process involved deduplication and the correcting of drug names to ensure inconsistency across the database. The role code primary suspect (PS) was appointed to the medication supposed most likely to lead to adverse events (AEs). Figure 1 details the data retrieval process from 2014Q1 to 2024Q3. After removing the duplicates, 2,634 reports were selected for the analysis of AEs.

### Disproportionality analysis

Disproportionality assessments were performed using the reporting odds ratio (ROR) and information component (IC), which offered similar outcomes. The signal was considered to be significant when IC and IC025 > 0 (46) and ROR and lower limit of 95% CI > 1 in no less than three patients (47). ROR is a frequentist method (non-Bayesian), with relatively low specificity and false positives. A higher ROR value indicates a stronger association. Meanwhile, IC is a Bayesian method, which guaranteed specificity and accuracy by considering the unexpectedly high risk of adverse reactions and decreasing the false positives (48). The classification of reports is provided in Table 1.

**Table 1:** Equations and criteria for ROR and IC.

Algorithm	Equation	Signal detection criteria
ROR	ROR = $\frac{ad}{bc}$	*a* ≥ 3, lower limit of 95% CI > 1
	95% CI = $e^{\ln\left(\text{ROR}\right)\;\pm \;1.96\sqrt{\frac{1}{a}\;+\;\frac{1}{b\;}\;+\;\frac{1}{c}\;+\;1/d}}$	
IC	IC = $\log_{2}a\left(a\;+b\;+\;c\;+\;d\right)/\left(a+\;c\right)\left(a+\;b\right)$	IC025 > 0
	IC025 = IC − 2 SD	

Note: ‘*a*’ represents the number of patients experiencing target drug-specific adverse reactions, ‘*b*’ pertains to those with other adverse reactions related to the target drug, ‘*c*’ corresponds to patients with adverse reactions related to other drugs not involving the target drug, and ‘*d*’ denotes those with other adverse reactions not related to the target drug.

The ROR of depression and suicide/self-injury associated with the combination of GLP-1RAs and metformin was contrasted to the ROR of the same issue associated with GLP-1RAs alone, which was employed as the control. For this study, ethical approval was not required because of the anonymity of data.

### Time‐to‐onset (TTO)

As is defined to be the time interval between the initial administration of the drug and the first documented occurrence of depression or suicide/self-injury, TTO was assessed by median with interquartile range and the Weibull shape parameter (WSP) test. We evaluated the reports that TTO was available. The occurrence rate of AEs was evaluated by the WSP. The shape parameter *β* determines the form of the distribution function and demonstrates the hazard. The random failure type is defined to be a constant hazard rate over time, mathematically characterized by *β* equal to or near 1 and its 95% CI includes 1. The early failure type is believed to be a decreased hazard rate over time, which is featured by *β* < 1 and its 95% CI < 1. The wear-out failure type is estimated as increased hazard rate over time, mathematically featured by *β* > 1 and 95% CI excludes 1 (49).

### Statistics analysis

We report non-normally distributed variables as median (IQR). Binary variables are presented as frequency and percentage. We evaluated the risk of depression and suicide/self-injury in patients treated with GLP-1RAs combined with metformin and GLP-1RAs alone by plotting the Pearson chi-squared (*χ*^2^) or Fisher’s exact test, with *P* < 0.05 considered to be significantly different. Subgroup analyses were conducted, stratified by gender and antidepressant usage, to examine individual characteristics in greater depth. R Studio 4.3 was conducted to analyze the data and represent the graph.

## Results

### Literature search and baseline characteristics

During the 10-year research period, a total of 2,634 reports associated with the combination of GLP-1RAs and metformin and 40,841 reports associated with the GLP-1RAs monotherapy were documented. There were 597 and 579 reports of depression (except suicide and self-injury) and suicide/self-injury, respectively, of which 47 and 104 reports were related to the combination therapy, separately.

The characteristics of the reports involved are listed in Table 2. In the reports of depression (except suicide and self-injury), there were more females with 370 (67.3%) reports in the GLP-1RAs monotherapy and 31 (66.0%) reports in the combination therapy of GLP-1RAs and metformin, with no significance found (*P* = 0.098). Within the cohort of GLP-1RAs-associated suicide/self-injury, 267 (56.2%) were female and 179 (37.7%) were male. However, in the cohort of GLP-1RAs combined with metformin, there are more males (59.6%) than females (35.6%) at risk of suicide/self-injury (*P* < 0.001). Regarding age, more patients were under 64 years old (*n* = 635, 54.0%), with 187 patients (15.9%) above 64. In regard to report source of depression (except suicide and self-injury), more reports were focused on the customers, with 386 (70.2%) reports in GLP-1RAs and 33 (70.2%) reports in the combination group. As regards suicide/self-injury, more reports were provided by the customers, with 253 (53.3%) in the GLP-1RAs group and 39 (37.5%) in the combination group of GLP-1RAs and metformin.

**Table 2:** Characteristics of reports with GLP-1RAs and metformin in depression and suicide/self-injury.

	Depression (except suicide and self-injury)			Suicide/self-injury		
	GLP-1RA (*n* = 550) (*n*, %)	Combined treatment (*n* = 47) (*n*, %)	*P*	GLP-1RA (*n* = 475) (*n*, %)	Combined treatment (*n* = 104) (*n*, %)	*P*
Sex						
Female	370 (67.3%)	31 (66.0%)	0.098	267 (56.2%)	37 (35.6%)	<0.001
Male	140 (25.5%)	16 (34.0%)		179 (37.7%)	62 (59.6%)	
Unknown	40 (7.3%)	0		29 (6.1%)	5 (4.8%)	
Weight						
<50 kg	0	0		2 (0.4%)	2 (1.9%)	
50–100 kg	137 (24.9%)	19 (40.4%)		127 (26.7%)	37 (35.6%)	
>100 kg	66 (12.0%)	11 (23.4%)		70 (14.7%)	12 (11.5%)	
Unknown	347 (63.1%)	17 (36.2%)		276 (58.1%)	53 (51.0%)	
Age (years)						
18–64	279 (50.7%)	26 (55.3%)		277 (58.3%)	53 (51.0%)	
>64	85 (15.4%)	11 (23.4%)		62 (13%)	29 (27.9%)	
Unknown	186 (33.8%)	10 (21.3%)		136 (28.6%)	22 (21.2%)	
Report source						
Customer (CN)	386 (70.2%)	33 (70.2%)		253 (53.3%)	39 (37.5%)	
Healthcare professional (HP)	56 (10.2%)	4 (8.5%)		57 (12.0%)	10 (9.6%)	
Physician (MD)	75 (13.6%)	7 (14.9%)		135 (28.4%)	38 (36.5%)	
Pharmacist (PH)	21 (3.8%)	2 (4.3%)		19 (4.0%)	10 (9.6%)	
Other healthcare professional (OT)	3 (0.5%)	1 (2.1%)		7 (1.5%)	7 (6.7%)	
Unknown	9 (1.6%)	0 (0%)		4 (0.8%)	0 (0%)	
Serious outcome						
Death (DE)	9 (1.6%)	1 (2.1%)	0.562	59 (12.4%)	40 (38.5%)	<0.001
Disability (DS)	34 (6.2%)	5 (10.7%)	0.379	23 (4.9%)	3 (2.9%)	0.541
Hospitalization – initial or prolonged (HO)	37 (6.7%)	5 (10.6%)	0.478	34 (7.2%)	14 (13.5%)	0.035
Life-threatening (LT)	45 (8.2%)	3 (6.4%)	0.908	72 (15.2%)	11 (10.6%)	0.227
Other serious (OT)	163 (29.6%)	20 (42.6%)	0.065	211 (44.4%)	29 (27.9%)	0.002
Missing	262 (47.6%)	13 (27.7%)		76 (16.0%)	7 (6.7%)	

Note: *n*, number of AEs reported; *P* < 0.05, statistical significance.

**Figure 2:**
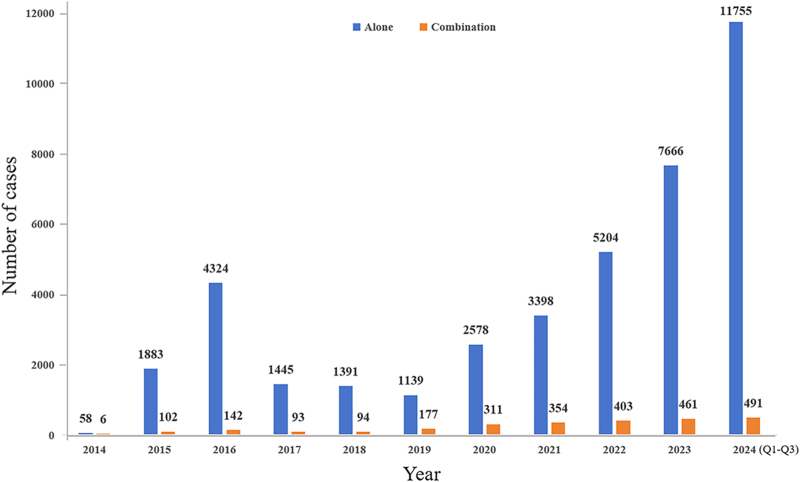
Reporting year of data.

It is noteworthy that the mortality attributed to suicide/self-injury was documented in 40 patients (38.5%) treated with the combination of GLP-1RAs and metformin, which is significantly higher than the GLP-1RAs monotherapy (12.4%) (*P* < 0.001). Moreover, a higher mortality due to depression (except suicide and self-injury) was found in the combination therapy than the GLP-1RAs monotherapy (2.1 vs 1.6%) although no significance was found (*P* = 0.562). In regard to the occurrence of hospitalization – initial or prolonged attributed to suicide/self-injury, more cases were found in the combination therapy of GLP-1RAs and metformin when compared with the GLP-1RAs monotherapy (13.5 vs 7.2%, *P* = 0.035). A higher risk of hospitalization – initial or prolonged in depression (except suicide and self-injury) was found in the combination therapy than in the GLP-1RAs monotherapy (10.6 vs 6.7%) although no significance was found (*P* = 0.478). The AEs of depression and suicide/self-injury associated with GLP-1RAs combined with metformin and the GLP-1RAs monotherapy were reported during the Q1–Q3 of 2024 (Fig. 2).

### Disproportionality analysis

**Figure 3:**
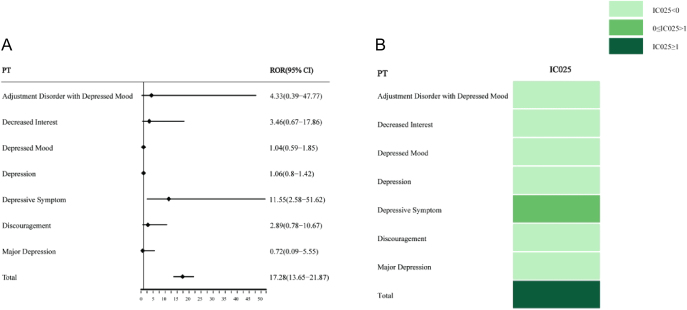
Disproportionality analysis for depression (except suicide and self-injury) related to GLP-1RAs + metformin versus GLP-1RAs at PT levels. (A) ROR; (B) IC.

**Figure 4:**
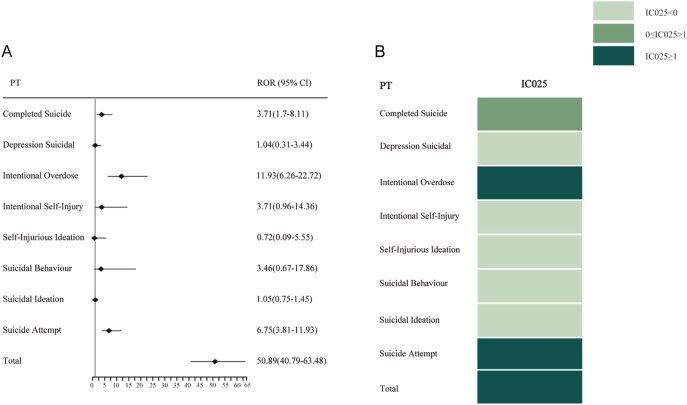
Disproportionality analysis for suicide/self-injury related to GLP-1RAs + metformin versus GLP-1RAs at PT levels. (A) ROR; (B) IC.

As reported in Fig. 3, disproportionality analysis using ROR and IC values showed that the combination of metformin and GLP-1RAs was found to be related to the higher incidence of depression (except suicide and self-injury) (ROR = 17.28, 95% CI: 13.65–21.87; IC = 3.98, IC025 = 3.64) when compared to the GLP-1RAs monotherapy. Each PT of depression (except suicide and self-injury) with GLP-1RAs combined with metformin was calculated using the disproportionality analysis. When compared with the GLP-1RAs monotherapy, a higher risk of depressive symptoms was revealed in the combination therapy (ROR = 11.55, 95% CI: 2.58–51.62; IC = 2.46, IC025 = 0.82). The results of the disproportionality analysis performed are shown in Fig. 4. The results show that a higher risk of suicide/self-injury (ROR = 50.89, 95% CI: 40.79–63.48; IC = 5.32, IC025 = 5) was found in GLP-1RAs combined with metformin compared to the GLP-1RAs monotherapy. Then, each PT of suicide/self-injury with GLP-1RAs combined with metformin was calculated using the disproportionality analysis. Compared with the GLP-1RAs monotherapy, significantly higher safety signals were detected in the combination therapy, including completed suicide (ROR = 3.71, 95% CI: 1.70–8.11; IC = 1.54, IC025 = 0.49), intentional overdose (ROR = 11.93, 95% CI: 6.26–22.72; IC = 2.48, IC025 = 1.72), and suicide attempt (ROR = 6.75, 95% CI: 3.81–11.93; IC = 2.08, IC025 = 1.34).

Considering the use of concomitant antidepressants (e.g., benzodiazepines, antidepressants, antipsychotics, opioids, sedatives, GABAergic drugs, and other psychoactive substances), we conducted an unmasking analysis (all cases in which concomitant medicines were identified were excluded here). After unmasking, GLP-1RAs combined with metformin were also found to be associated with higher depression than the GLP-1RAs monotherapy (ROR = 22.38, 95% CI: 15.83–31.65), which was similar to those with antidepressants. Similarly, the unmasking analysis on the risk of suicide/self-injury was performed. The combination of GLP-1RAs and metformin was found to be associated with a higher risk of suicide/self-injury when compared with the GLP-1RAs monotherapy (ROR = 52.11, 95% CI: 40.08–67.73), which have similar outcomes with those containing antidepressants.

Considering the use of concomitant antidiabetic agents (e.g., SGLT-2 inhibitors, DPP-4 inhibitors, thiazolidinediones, alpha-glucosidase inhibitors, and sulfonylureas), we performed an unmasking analysis (all cases in which concomitant medicines were identified were excluded here). After unmasking, the combination of GLP-1RAs and metformin was confirmed to be related to a significantly higher risk of depression than GLP-1RAs alone (ROR = 39.7, 95% CI: 25.96–60.72), which was consistent with those with antidiabetic drugs. In addition, the unmasking analysis on the risk of suicide/self-injury was conducted. GLP-1RAs combined with metformin were associated with an increased risk of suicide/self-injury compared with the GLP-1RAs monotherapy (ROR = 68.13, 95% CI: 52.07–89.14), which have similar outcomes with those containing antidiabetic drugs.

**Figure 5:**
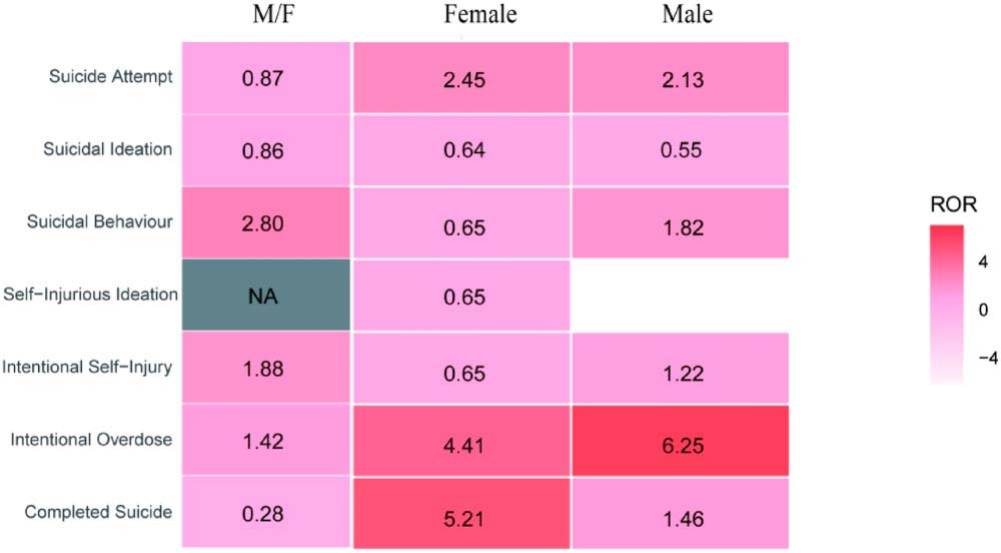
Heat maps displaying sex-specific RORs and the ratios of these RORs on the risk of suicide/self-injury in GLP-1RAs + metformin vs GLP-1RAs.

A subgroup analysis stratified by sex on the risk of suicide/self-injury was performed. Figure 5 illustrates RORs for female, male, and the ratio of male to female. Overall, RORs for suicide/self-injury risk in men were generally comparable to or higher than those in women. RORs for suicidal behavior, intentional self-injury, and intentional overdose were 2.80, 1.88, and 1.42 times higher in male than in female, respectively. However, RORs for completed suicide were 0.28 lower in male than in female.

### Analysis of TTO

**Figure 6:**
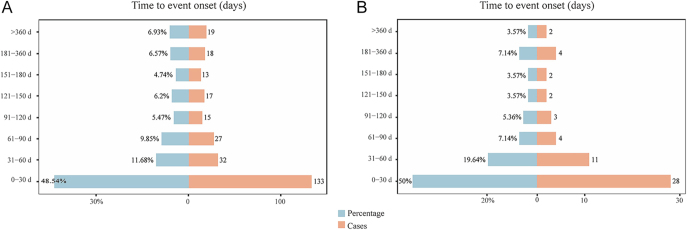
TTO of depression and suicide/self-injury among GLP-1RAs and their combination therapy. (A) GLP-1RAs monotherapy; (B) GLP-1RAs + metformin.

**Figure 7:**
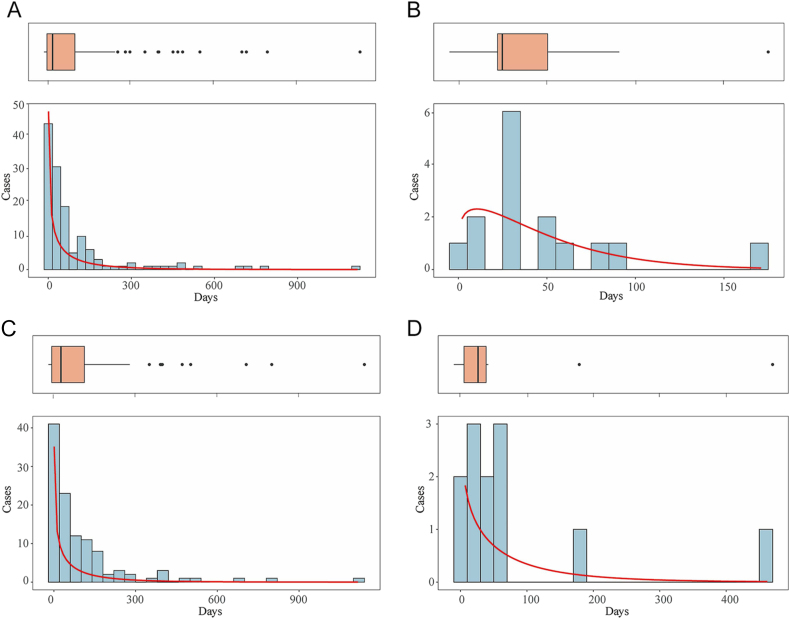
Histogram of depression and suicide/self-injury. (A) Depression (except suicide and self-injury) in GLP-1RAs monotherapy, (B) depression (except suicide and self-injury) in GLP-1RAs + metformin, (C) suicide/self-injury in GLP-1RAs monotherapy, and (D) suicide/self-injury in GLP-1RAs + metformin.

The occurrence of AEs in different groups for suicide/self-injury and depression is shown in Fig. 6. It presents the distribution of AEs onset time, with reports indicating that the majority of AEs, at rates of 48.54% in GLP-1RAs and 50% in the combination of GLP-1RAs and metformin, respectively, occurred during the first month of medication. AEs reached their lowest incidence at 3–6 months but rose after 6 months (13.5 and 10.71%). The results of the WSP test and histograms are presented in Fig. 7. Our data revealed that patients treated with GLP-1RAs combined with metformin reported a shorter median time of suicide/self-injury (41.5 (21.5–53) vs 45 (12.5–127.5), *P* = 0.67) and depression (except suicide and self-injury) (30 (27.50–54.00) vs 31 (11.25–109.75), *P* = 0.34) than those treated with GLP-1RAs alone, although no significance was detected. In addition, a shorter median time of suicide/self-injury (41.5 (21.5–53) vs 58 (5.50–69)) and depression (except suicide and self-injury) (30 (27.50–54.00) vs 32 (17.50–88.50)) was found in the combination therapy of GLP-1RAs and metformin compared to treatment with metformin alone.

Results of WSP analyses for GLP-1RAs associated with suicide/self-injury showed that the shape parameter *β* was <1 and its 95% CI < 1, which demonstrated an early failure type. Conversely, the results of GLP-1RAs combined with metformin associated with suicide/self-injury showed *β* of 0.81 with its 95% CI including 1, implying a random failure type profile. When considering depression (except suicide and self-injury), patients treated with the GLP-1RAs monotherapy exhibited an early failure type, while the combination therapy of GLP-1RAs and metformin demonstrated a random failure type profile (Fig. 7, Table 3).

**Table 3:** Weibull shape parameter test for depression and suicide/self-injury associated with GLP-1RAs versus GLP-1RAs and metformin.

Database	Case reports	Median (*d*) (25–75%)	Scale parameter: *α* (95%CI)	Shape parameter: *β* (95%CI)	Type
Suicide/self-injury					
GLP-1RA	111	45 (12.5–127.5)	79.63 (57.06–102.21)	0.69 (0.60–0.79)	Early failure type
Metformin	34	58 (5.50–69)	92.40 (22.14–162.66)	0.47 (0.36–0.58)	Early failure type
Combined treatment	12	41.5 (21.5–53)	70.21 (18.24–122.17)	0.81 (0.48–1.14)	Random failure type
Depression (except suicide and self-injury)					
GLP-1RA	134	31 (11.25–109.75)	71.81 (52.74–90.89)	0.68 (0.59–0.76)	Early failure type
Metformin	43	32 (17.50–88.50)	98.04 (44.67–151.41)	0.58 (0.46–0.71)	Early failure type
Combined treatment	15	30 (27.50–54.00)	49.42 (27.24–71.61)	1.19 (0.73,1.65)	Random failure type

## Discussion

The neuropsychiatric safety of GLP-1RAs combined with metformin has caused concern, largely attributed to their synergistic central appetite-suppressing effects. Mounting concerns have emerged regarding suicidal/depression risk associated with semaglutide, including its use in combination therapy with metformin. Multiple articles reported the link between GLP-1RAs and the occurrence of depression and suicide/self-injury. However, the suicide/self-injury risk profile of GLP-1RAs combined with metformin has never been examined, although this regime is recommended as a first-line therapy for T2DM. This study provides the first comparative analysis of depression and suicide/self-injury risks between the combination therapy of GLP-1RAs and metformin and the GLP-1RAs monotherapy.

GLP-1RAs are usually used in combination with metformin in T2DM. The guidelines recommended metformin as the first-line treatment in lowering the glucose and that metformin be maintained as part of T2DM treatment if no contraindications are present. Recently, the emphasis of T2DM has been transferred from primarily low glucose to low glucose with CV–renal risk (18). GLP-1RAs had been recommended as the first-line treatment for diabetic patients with CV–renal risk, in the presence of metformin as the first choice for the combination. However, the majority of phase III clinical trials and CVOTs of GLP-1RAs were performed in diabetes administrated with metformin. Therefore, it is impossible to accurately deduce the CV–renal protective profile of GLP-1RAs in the absence of metformin. Moreover, the ESC guideline recommended the addition of GLP-1RAs, with the treatment of metformin for individuals at ASCVD or high/very high CV risk (7).

**Figure 8:**
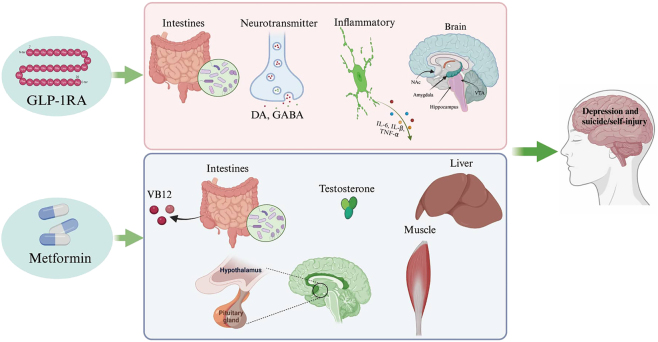
Mechanism of depression and suicide or self-injury.

Our results showed a significant risk profile for suicide/self-injury linked to GLP-1RAs, demonstrating higher incidence in females than in males. However, the addition of metformin reversed the phenomenon. The suicide/self-injury related to males is considered higher compared with females in the combination therapy of GLP-1RAs and metformin. The results above demonstrated that, compared with females, the addition of metformin to GLP-1RAs exhibited a significantly stronger association with an elevated risk of suicide/self-injury in males. The mechanism may be attributed to the regulation action of metformin of the HPA axis and testosterone (see Fig. 8). A clinical analysis encompassing 70 diabetic male patients revealed that metformin could decrease the testosterone level of males (50), the primary androgen responsible for regulating male external genitalia development and secondary sexual characteristics. Another cross-sectional study replicated these findings, demonstrating consistent results regarding the depressive action of metformin on the level of testosterone (51). Furthermore, compared to the GLP-1RAs monotherapy, the mortality and hospitalization – initial or prolonged attributed to suicide/self-injury were significantly higher in the combination therapy of GLP-1RAs and metformin. This confirmed that an individual using both metformin and GLP-1RAs concurrently is at an increased risk of severe ADR than monotherapy, which should be watched closely.

Current proofs regarding the association between GLP-1RAs and suicidal risk remain inconclusive. Chen *et al.* demonstrated the absence of any safety signals in suicide/self-injury due to GLP-1RAs, with a marginally elevated risk of RORs observed in children (45). Conversely, a lower occurrence of suicidal ideation or attempts was observed in obese teenagers treated with GLP-1RAs than those who underwent lifestyle interventions (52). However, Guirguis discovered that the safety signal of suicide/self-injury related to semaglutide and tirzepatide exhibited distinct characteristics; thus, the causal link between GLP-1RAs and suicide/self-injury cannot be concluded (53). Previously, people tended to focus on the potential risk of suicide/self-injury in the GLP-1RAs monotherapy, while overlooking the critical therapeutic implications of metformin combination therapy in T2DM.

As the cornerstone and first-line therapeutic agent for T2DM, metformin ameliorates glucolipid metabolism by enhancing the insulin sensitivity, suppressing hepatic gluconeogenesis, and reducing the absorption of intestinal glucose. In our study, the addition of metformin may elevate suicide/self-injury risk linked to GLP-1RAs. Guirguis *et al.* exposed the higher suicide/self-injury risk in patients who received metformin when compared to those receiving semaglutide, tirzepatide, and liraglutide (53), which was in alignment with our outcome. Furthermore, our study observed that the addition of metformin significantly elevated the incidence of completed suicide, intentional overdose, and suicide attempt, which were categorized as the PT of suicide/self-injury, when compared with the GLP-1RAs monotherapy. The outcome implied that, although no significant risk of suicide/self-injury was found in the GLP-1RAs monotherapy, the addition of metformin warrants close attention based on the available data.

GLP-1RAs, with improving body image, may contribute to the enhancement of self-esteem, positively influencing the psychological health of many individuals. Current evidence has remained inconsistent conclusions regarding the relationship between GLP-1RAs and depression risk. A meta-analysis confirmed that patients treated with GLP-1RAs were associated with significant antidepressant effects (54). However, case reports documented the emergence of depressive symptoms linked to semaglutide in patients with and without pre-existing depression disorder, thereby demonstrating the potential impact of GLP-1RAs on mood (55, 56). The mechanism may be attributed to the modulation of GLP-1RAs on the central nervous system. Through activating the GLP-1 receptors in brain, GLP-1RAs exert potent anorexigenic effect. As is known, the consumption of palatable high-sugar and high-fat foods may stimulate the mesolimbic dopamine pathway, producing sensations of pleasure and satiety. GLP-1RAs could restrain the reward feedback, diminish the neural basis of pleasure, and cause a profound reward deficit, which is the core symptom of depression.

Compared to the GLP-1RAs monotherapy, the assessment of depression risk in the combination of GLP-1RAs and metformin warrants greater attention. The underlying mechanism may involve the regulation action of metformin of the HPA axis and testosterone (see Fig. 8). As the first-line oral antidiabetic drug, metformin decreases the level of blood glucose and lipid and largely modulates the metabolic disorder in T2DM. During 2020, metformin prescriptions in the US exceeded 92 million for conditions related to impaired glucose metabolism. A prospective observational study, involving 120 subjects, reported that metformin may induce depression and reduce the quality of life in T2DM; thus, the assessment of depression is needed in patients using metformin (57). Moore *et al.* found an increased risk of cognitive impairment in T2DM patients treated with metformin. In addition, the impact of metformin on cognitive performance may be improved by the modulation of serum vitamin B12 concentrations (58). In line with the above, in our results, the concomitant use of metformin was shown to increase the depression risk of GLP-1RAs, which is pioneering in exploring the association between the depression risk and the combination therapy of GLP-1RAs and metformin. In agreement with our findings, the addition of metformin was reported to enhance the risk of depression associated with sitagliptin, reversing the antidepressant effects observed in sitagliptin alone (59, 60). The mechanism may be attributed to the regulation of vitamin B12 by metformin, whose deficiency has emerged as a recognized issue among diabetic patients receiving this therapy (61, 62, 63, 64), which potentially occurred after 4–5 years of continuous treatment. Interestingly, the deficiency of vitamin B12 was associated with a raised depression risk, highlighting the necessity of supplementation on ameliorating mood (65).

However, the role of metformin in the risk of depression is still not mixed. Some studies reported that metformin may have benefits in neurological diseases. However, its antidepressant efficacy remains controversial. Chen *et al.* confirmed that metformin could decline the risk of depression in old men with T2DM (66). A study from Denmark confirms lower depression risks in patients with more prescriptions of metformin (67). However, the correlation should be interpreted with caution because the data derived from the Danish Register were of retrospective nature and there is lack of information on dose and treatment adherence. Furthermore, in a previous study, the symptom of depression in the metformin group was not found to show more amelioration than in controls (68).

TTO analysis was conducted to examine the onset profiles of depression (except suicide and self-injury) and suicide/self-injury in patients administered GLP-1RAs and metformin. The median TTO of depression (except suicide and self-injury) and suicide/self-injury was shorter in patients administrated with the combination of GLP-1RAs and metformin than the GLP-1RAs or metformin monotherapy, although the difference was not significant. This confirmed that combination-associated depression (except suicide and self-injury) and suicide/self-injury exhibited a shorter therapy duration than depression (except suicide and self-injury) and suicide/self-injury linked to monotherapy. Notably, the majority of cases were found to occur within the first month of treatment (combination therapy vs monotherapy: 50 vs 48.54%).

The WSP analysis showed that GLP-1RAs or metformin monotherapy-associated depression (except suicide and self-injury) and suicide/self-injury exhibited an early failure-type pattern, highlighting the necessity for early detection. This demonstrated that the risk of depression (except suicide and self-injury) and suicide/self-injury linked to monotherapy was elevated in early treatment and subsequently reduced over time. The WSP results indicated the importance of initiating psychiatric monitoring early in the treatment, allowing for timely detection and intervention. In accordance with our results, Shu *et al.* revealed that the ADR, such as gastrointestinal AEs, linked to GLP-1RAs, was dose dependent and tended to diminish over time (69). Conversely, the combination of GLP-1RAs and metformin associated with depression (except suicide and self-injury) and suicide/self-injury had a random failure-type pattern, implying that the occurrence of depression (except suicide and self-injury) and suicide/self-injury linked to the combination therapy will persistently occur.

Although our findings confirmed a link between the intake of GLP-1RAs combined with metformin and an increased incidence of suicide/self-injury and depression than the GLP-1RAs monotherapy, it is crucial to recognize that disproportionality analysis is not independent, and security declaration should not be deduced. Moreover, it is essential to highlight that our analysis should not be interpreted as causation but rather emphasizing a statistical link, while acknowledging the limitations of disproportionality.

This study had several limitations. First, only spontaneous cases were reported in the FAERS database, without undergoing systematic validation. It is likely that the reports may be associated with the medical and psychiatric histories and unquantified illicit medication ingestion of the patients. Thus, the lack of contextual elements hindered the establishment of a causal relationship. Second, despite the higher incidence of depression and suicide/self-injury for the GLP-1RAs monotherapy, the number was greatly small for the combination of GLP-1RAs and metformin, which limits the stability and reliability of the estimate. The reports of FAERS were anonymized, which may lead to duplicate submissions. Third, the FAERS database is dominated by inherent reporting bias and incomplete data that could not document the change of medication, such as dose modifications or newly developed comorbidities. A further prospective study should be performed to distinguish between genuine safety signals and spurious findings arising from methodological limitations. Furthermore, a subgroup analysis stratified by different GLP-1RAs on the risk of suicide/self-injury or depression is imperative. Owing to the reports of FARES being retrospective, it should be treated with caution in establishing the causal relationship between drugs and depression and suicide/self-injury. The results should be interpreted as indicative of relevance rather than causality.

## Conclusion

The advantage of this analysis resides in its novelty, as this appears to be the first disproportionality assessment to investigate the safety profile of GLP-1RAs combined with metformin in T2DM patients compared with the GLP-1RAs monotherapy. Our results found that the combination of GLP-1RAs and metformin displayed a higher risk of completed suicide, intentional overdose, suicide attempt, and depressive symptoms compared with GLP-1RAs, which may inform clinical decision. However, a large-scale prospective investigation needs to be conducted to further confirm the mental health risk of the combination of GLP-1RAs and metformin, especially when metformin is added to GLP-1RAs therapy, particularly in relation to the mechanistic investigations.

## Declaration of interest

The authors declare that there is no conflict of interest that could be perceived as prejudicing the impartiality of the work reported.

## Funding

This work did not receive any specific grant from any funding agency in the public, commercial, or not-for-profit sector.

## Author contribution statement

Wenjun Ji wrote the original draft; Ying Ma analyzed the data and performed the database search; Linwei Chen revised and edited the manuscript; Jie Fang designed the protocols; and all authors read and approved the final manuscript.

## Data availability

The datasets generated and/or analyzed during the current study are available from the corresponding author on reasonable request.
